# Automated HPLC monitoring of broth components on bioreactors

**DOI:** 10.1155/S1463924689000532

**Published:** 1989

**Authors:** Eric Favre, Patrick Pugeaud, Jean Philippe Raboud, Paul Péringer

**Affiliations:** EPFL IGE-Laboratoire de Génie Biologique Lausanne CH-1015 Switzerland

## Abstract

Under proper operating conditions, a low dead volume continuous filtration module operated on biological broths (yeast and bacteria suspensions in stirred reactors) still fulfills the flow-rate requirements of an analytical apparatus (for example HPLC or FIA) without membrane regeneration. The filtrate stream has been successfully connected to a bioreactor in order to perform the automated HPLC analysis of broth components. The monitoring of the carbon source (lactose), and minor products (glycerol, acetate and succinate) during a yeast culture (Kluyveromyces marxianus) is shown.


Journal of Automatic Chemistry Vol. 11, No. 6 (November–December 1989), pp. 280–283

### Automated HPLC monitoring of broth components on bioreactors

**Eric Favre, Patrick Pugeaud, Jean Philippe Raboud and Paul Péringer**

*EPFL, IGE-Laboratoire de Génie Biologique, CH-1015 Lausanne, Switzerland*

*Under proper operating conditions, a low dead volume continuous filtration module operated on biological broths (yeast and bacteria suspensions in stirred reactors) still fulfills the flow-rate requirements of an analytical apparatus (for example HPLC or FIA) without membrane regeneration. The filtrate stream has been successfully connected to a bioreactor in order to perform the automated HPLC analysis of broth components. The monitoring of the carbon source (lactose), and minor products (glycerol, acetate and succinate) during a yeast culture* (Kluyveromyces marxianus) *is shown.*

### Introduction

The control and optimization of fermentation processes is mainly limited by the lack of adequate on-line sterilizable sensors for chemical parameters. In fact, *in situ* probes, using immobilized enzymes or antibodies, rarely show long-term stability, and are inactivated by steam sterilization [1]. These drawbacks can be overcome when a cell free stream, from the bioreactor or an external device, is connected to a probe, or analytical apparatus [2, 3].

Several attempts, using either dialysis [4] or membrane filtration [5], have shown limited life-time efficiency due to membrane fouling; steam-sterilizable systems with an automation facility are still lacking [6]. One possible strategy to achieve this, by using an ultrafiltration membrane and a low dead-volume continuous filtration module, has been investigated.

### Materials and methods

### *Filtration loop*

A home-made continuous flat filtration module (material: PVDF, 2 mm diameter spiral channel, membrane surface area 43 cm$^2$), was used. The internal diameter of the module (90 mm) was chosen so that a wide choice of disc membranes could be used. For the on-line analysis, the module was equipped with a 100 000 MWt cut-off membrane (YM100A membrane from Amicon, Danvers, Massachusetts, USA), on a porous sintered stainless-steel support (IC90 from Poral, Le Pont de Claix, France). Preliminary experiments showed better filtration efficiency when a pulsed feed stream was generated [7]; this was achieved by a positive displacement pump (CFG Prominent E0407 from Prominent GmbH, Heidelberg, F.R. Germany), equipped with a stream-sterilizable head. Before each experiment, the whole filtration loop (pump, module and membrane) connected by PFTE tubings (2 mm internal diameter) was steam sterilized and connected aseptically to the bioreactor. The dead volume of the whole filtration loop was less than 9 ml.

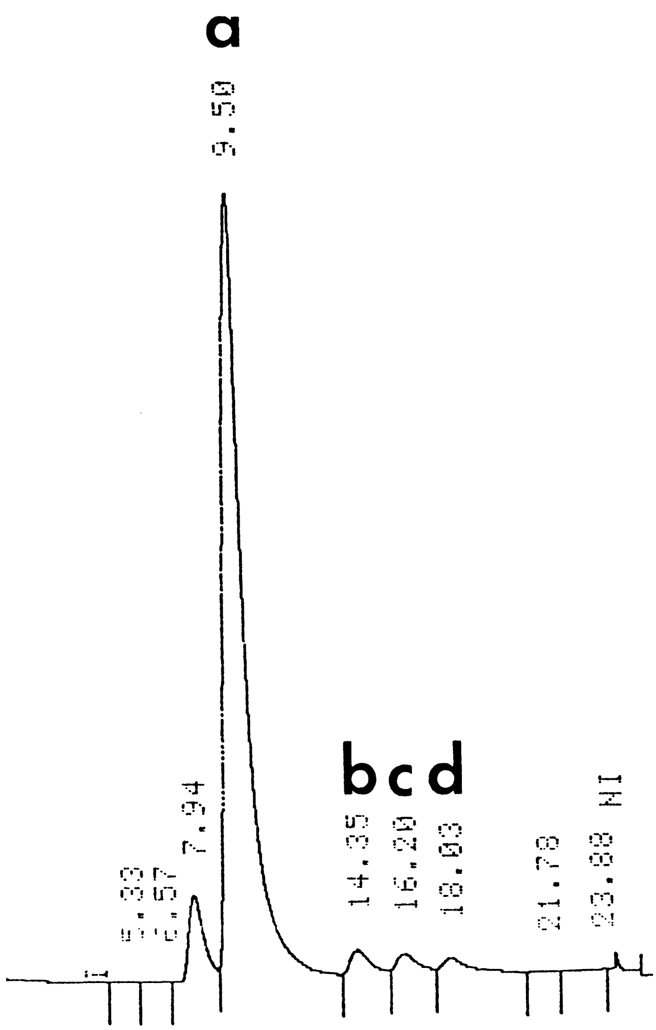

*Figure 1. Typical chromatogram obtained with an on-line filtrated sample: a = lactose, b = succinate, c = glycerol, d = acetate.*

### *HPLC analysis*

HPLC analysis was performed with a 2010 Varian isocratic pump (Varian Internationl AG, Basel, Switzerland), a VICI electrically actuated injection valve (Valco Europe, Schenkon, Switzerland), a Biorad HPX 87H column (Biorad, Richmond, Virginia, USA) thermostated at 55 °C in an oven, an Ercatech 7512 RI detector (Ercatech AG, Bern, Switzerland) and a Perkin

0142–0453/89 $3.00 © 1989 Taylor & Francis Ltd.

Elmer LCI 100 integrator (Perkin Elmer AG, Küsnacht, Switzerland). The mobile phase was 0·01 N sulphuric acid, filtered on a 0·22 μm membrane, and continuously degassed with vacuum (Ercatech 3512 degasser). The method was calibrated before use (off line) by an external standard. The following variation coefficients were obtained on eight successive standard injections: lactose 0·25%; succinate 0·41%; glycerol 1·58%; acetate 2·16%. A typical chromatogram is shown in figure 1. For the automated sampling, an integrator relay actuated an electrical valve (Huba Control AG, Würenlos, Switzerland).

*Bioreactor and microbial cultures*

A 5 l working volume bioreactor (Bioengineering AG, Wald, Switzerland), with temperature, agitation, pH and dissolved oxygen control has been used. *Kluyveromyces*

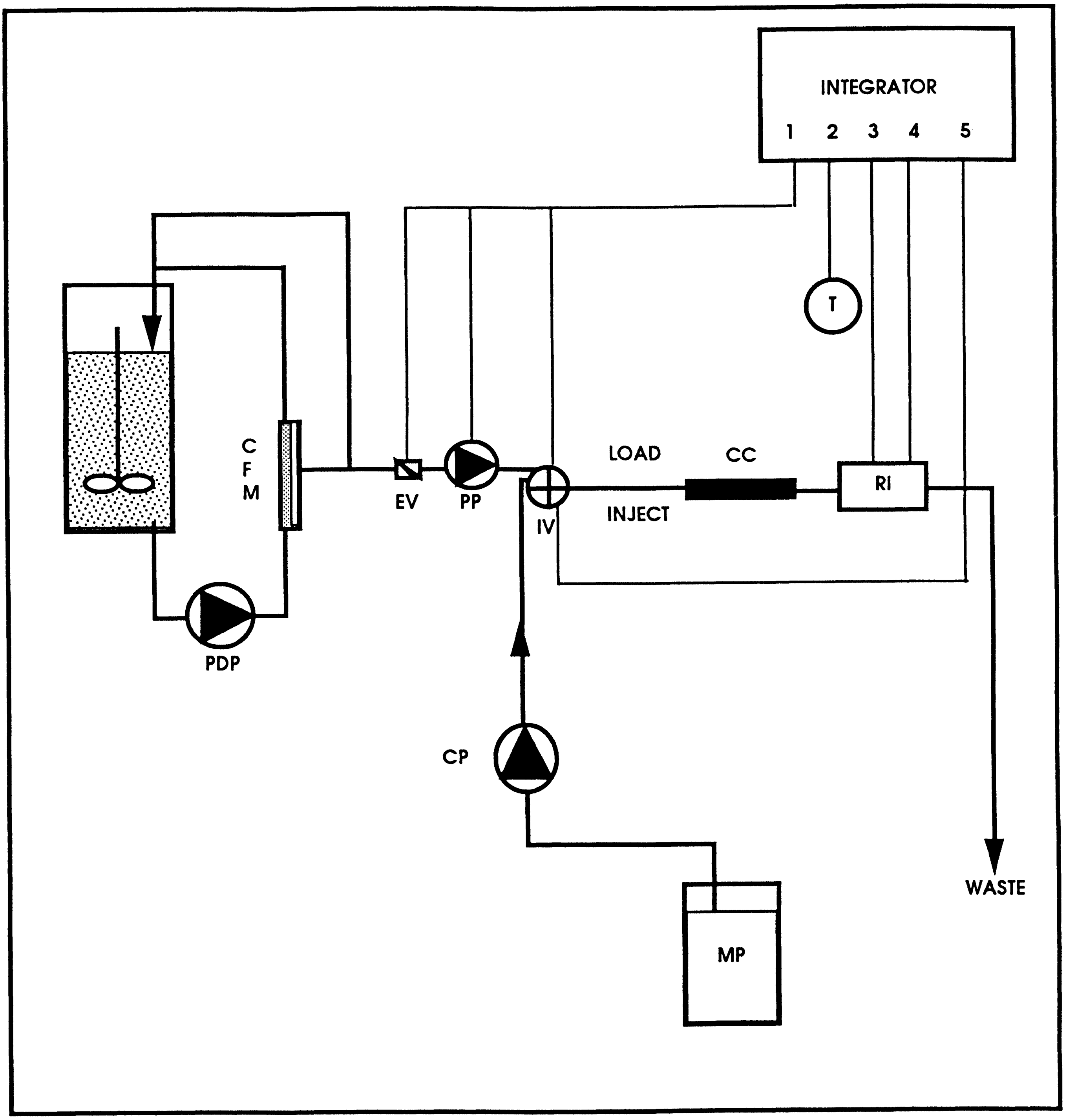

*Figure 2. Overall set-up and flowchart of the automated system. PDP, positive displacement pump; CFM, continuous filtration module; EV, electrical valve; PP, peristaltic pump; IV, injection valve; CP, chromatographic pump; MP, mobile phase; CC, chromatographic column; RI, refractive index detector; T, timer. Integrator relay numbers are detailed in the text.*

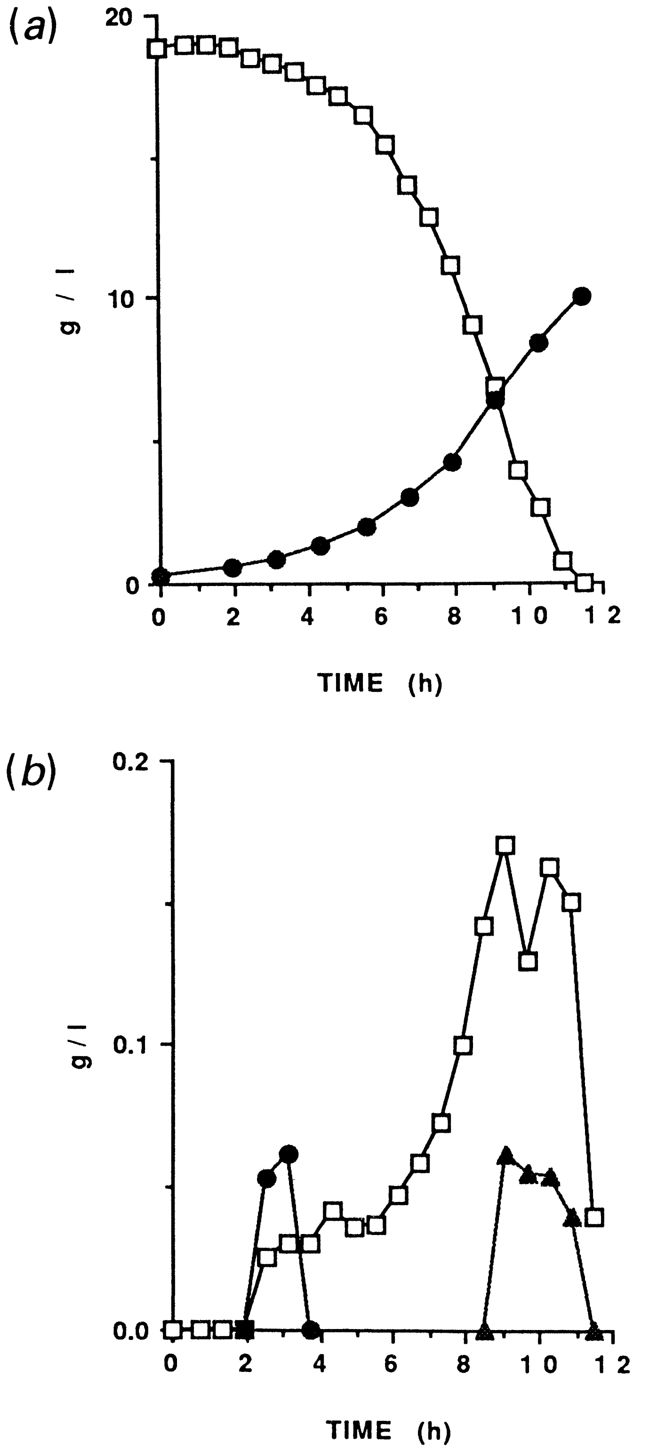

*Figure 3. Evolution of broth components concentrations during a yeast batch culture determined by automated HPLC.*
*(a) □, lactose; ●, yeast concentration determined off-line by dry weight measurement; (b) □, glycerol; ●, acetate; ▲, succinate.*

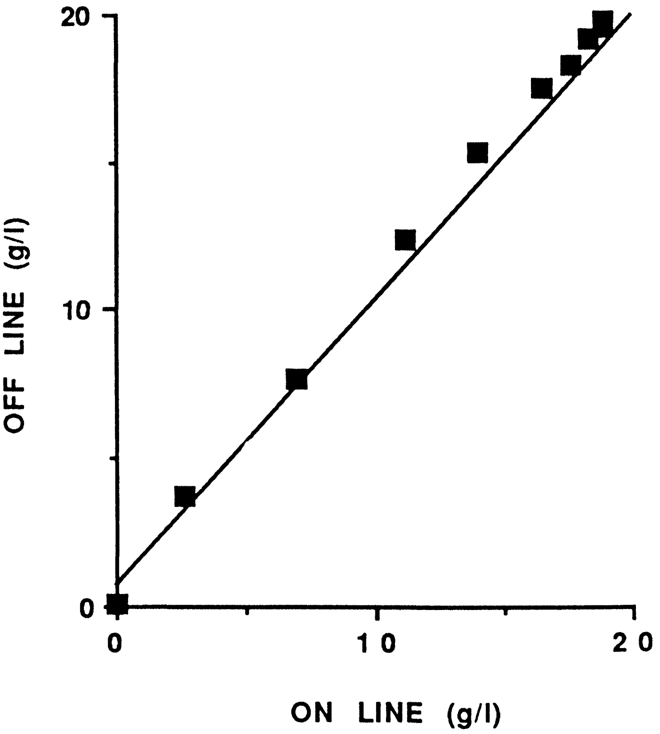

*Figure 4. Comparison of the lactose concentration determined by the on-line automated HPLC method and by the reference off-line method.*

*marxianus* strain (NCYC, Nutfield, Surrey, UK) was cultured on a semi-synthetic media (lactose 20 g/l, $[NH_4]_2SO_4$ 4 g/l, $KH_2PO_4$ 1 g/l, yeast extract 1 g/l) in batch and/or continuous modes in order to test the sterility containment of the filtration loop and check the performances of the analytical system. All reagents were of analytical grade from Sigma Co., St Louis, Missouri, USA.

### Experimental set-up

The overall-set-up is shown in figure 2. A bypass is installed on the filtrate tubing, and an electrical valve piloted by an integrator relay, is opened when analysis is desired. A lag time of 5 min is used in order to rinse thoroughly the filtrate flow-path (average filtrate flow-rate 0·5 to 2 ml/mn); the electrical injection valve is subsequently actuated and the run begins. A typical time chart of the integrator relays is shown in the table.

*Time chart of the integrator relays.*

| Event time (min) | Relay | State | Event function |
|---|---|---|---|
| 0·000 | 1 | Closed | Loading loop opened |
| 0·003 | 1 | Open | Re-actuating relay 1 |
| 1·500 | 2 | Closed | Analysis frequency timer actuated |
| 2·000 | 3 | Closed | RI detector purge |
| 2·020 | 3 | Open | Re-actuating relay 3 |
| 3·000 | 4 | Closed | RI detector auto zero |
| 3·020 | 4 | Open | Re-actuating relay 4 |
| 4·999 | Chart | On | Chart paper on |
| 5·000 | 5 | Closed | Injection valve actuated |
| 5·520 | 5 | Open | Re-actuating relay 5 |
| 6·500 | A/Z | On | Printer auto zero |

A maximum analysis frequency of 0·75 $h^{-1}$ can be obtained with this method.

### Results and discussion

Changes occuring during a batch culture of *Kluyveromyces marxianus* (figure 3) can be observed by the present automated method for the substrate (lactose) and three minor byproducts (glycerol, succinate, acetate). Figure 4 demonstrated that, in comparison with the off-line method, the automated method performed extremely well for lactose. The regression equation is $y = 1{\cdot}017x + 0{\cdot}670$, where $y$ represents the results obtained by the off-line method. The correlation coefficient is 0·998. Such a statistical analysis could not have been applied to the other compounds due to the limited number of off-line samples containing a significant amount of glycerol, acetate or succinate. The redundancy provided by the 'high' frequency of measurement (compared with off-line analysis performed generally no more frequently than every 4 h) enables the visualization of short and weak transitory metabolic changes (for example, acetate and succinate release), and add confidence to the subsequent statistical data processing (for example, calculation of kinetic constants).

Despite the raw pretreatment of the sample (one single ultrafiltration), and the numerous possible interferences (for example, bubbles, contamination in the tubing, and retention of molecules in the polarization gel of the membrane), the HPLC analysis was more than satisfactory. This filtration system has been successfully operated for more than 2 months during a continuous baker's yeast culture, without membrane regeneration. The automated system described should be very attractive when it is necessary to connect an analytical apparatus (with low sample volume requirements), or heat-sensible probes, to a bioreactor.

### References

1. Clarke, D., Blake-Coleman, B., Calder, M., Carr, R. and Moody, S., *Journal of Biotechnology,* **1** (1985), 135.
2. Chotani, G. and Constantidines, A., *Biotechnology and Bioengineering,* **24** (1982), 2743.
3. Dinwoodie, R. and Mehnert, D., *Biotechnology and Bioengineering,* **27** (1985), 1060.
4. Zabriskie, D. and Humphrey, A., *Biotechnology and Bioengineering,* **20** (1978), 1295.
5. Ghoul, M., Ronat, E. and Engasser, J. M., *Biotechnology and Bioengineering,* **28** (1986), 119.
6. Monseur, X. and Motte, J. C., *Analytica Chimica Acta,* **204** (1988), 127.
7. Bauser, H., Chmiel, H., Stroh, N. and Walitza, E., *Journal of Membrane Science,* **11** (1982), 321.

